# The my experience of taking medicines (MYMEDS) questionnaire for assessing medicines adherence barriers in post-myocardial infarction patients: development and utility

**DOI:** 10.1186/s12872-020-01362-y

**Published:** 2020-02-03

**Authors:** Rani Khatib, Nasrin Patel, Alistair S. Hall

**Affiliations:** 1 Medicines Management & Pharmacy Services, Leeds Teaching Hospitals NHS Trust, Leeds, UK; 2 Cardiology Department, Leeds Teaching Hospitals NHS Trust, Leeds, UK; 3 Leeds Institute of Cardiovascular and Metabolic Medicine, University of Leeds, Leeds, UK

**Keywords:** Cardiovascular diseases, Medicines adherence, Shared decision making, Myocardial infarction, Secondary prevention

## Abstract

**Background:**

The ‘My Experience of Taking Medicines’ (MYMEDS) questionnaire is a self-reporting tool for identifying modifiable adherence barriers among individuals prescribed post-myocardial infarction (MI) secondary prevention medicines (SPM) in clinical practice. It was found to be a useful tool to support the conduction of patient-centred consultation in cardiology outpatient leading to improved outcomes including better adherence to SPM and patient satisfaction. This study describes the rationale and development of the MYMEDS tool, its performance and usefulness in identifying modifiable barriers to adherence in cardiology medical practice including user feedback of 204 consecutive post-MI patients who completed an evaluation based on MYMEDS.

**Methods:**

Modifiable non-adherence factors were initially identified based on literature review and stakeholder feedback. A draft MYMEDS questionnaire was piloted in 10 patients and adapted accordingly. The final version comprises six sections, covering current medicines, understanding and satisfaction with medicines, concerns about medicines, practical adherence barriers, fitting medicines into daily routine, and adherence to individual SPMs. The questionnaire was mailed to post-MI patients who then attended an outpatient medicines optimisation clinic.

**Results:**

Mean age was 70.5 years and 67.6% were male. The tool was effective in revealing modifiable adherence barriers that could be addressed during the consultation. There were high rates of concern that SPMs could be harmful (33.2%) or overprescribed (43.2%), practical issues with swallowing medicines (8.2%), opening packaging (7.3%) or accessing repeat prescriptions (5.2%), forgetfulness (19.7%), and concerns about inconvenience (13.5%). Mean number of barriers per patient was 1.8 ± 1.5. The medications most commonly associated with non-adherence were statins (21.5%), angiotensin II receptor blockers (21.1%), and antiplatelet agents (18.5%). In total, 42.5% of patients acknowledged non-adherence behaviour. Patient feedback on MYMEDS was positive, with near-unanimous agreement that it was simple, clear and not too long, and that it enabled them to raise any concerns they had about their medicines. Patients reported that their individual medicines related needs were better addressed.

**Conclusions:**

MYMEDS is a practical tool that can successfully identify modifiable barriers to SPM adherence which can be addressed in a clinical setting. It can be easily rolled out in daily clinical practice to enable individualised person-centred medicines optimisation consultation.

## Background

Adherence issues are a major concern in cardiovascular (CV) medicine. Indeed, rates of non-adherence to secondary prevention medicines (SPMs) – such as antihypertensives, statins and antiplatelet agents – have been estimated at around 40% based on refill data, self-reports and direct measures [1, 2].

The effects on patient outcomes can be highly significant. In a meta-analysis of 44 prospective studies, the risk of all-cause mortality was reduced by 45 and 29% in patients with good adherence to statins and antihypertensive agents, respectively, compared to those with poor adherence [2]. In the post-myocardial infarction (MI) setting, adherence to SPMs has been shown to significantly decrease the risk of further major vascular events or revascularisation [3].

The underlying causes of non-adherence need to be disentangled and independently understood, if they are then to be addressed. However, these causes are often complex and multifactorial, and typically cannot be explained by fixed factors like sociodemographic status, personality traits, or disease type and severity [4]. Hence, the reasons for non-adherence should be explored at an individual level – particularly those that are modifiable and hence can be addressed in practice – and interventions should be tailored to the needs of the particular patient.

In many circumstances, the most effective method for examining adherence may be patient self-reporting. Although memory and social desirability biases can lead to overestimation of overall adherence, self-reported non-adherence is largely reliable [4–8]. Self-report tools are also pragmatic and inexpensive for use in everyday clinical practice [4].

Many such instruments have been developed and are widely used in various medical conditions; some of these have been applied in CV medicine. Examples include the Single Question (SQ) tool [9], the eight-item©Morisky Medication Adherence Scale (MMAS-8) [10], the Adherence Estimator™ (AE) [11], the Medication Adherence Report Scale (MARS) [12], and the Beliefs about Medicines Questionnaire (BMQ) [13].

These tools were designed primarily to ‘detect’ non-adherence behaviour (e.g. SQ, MMAS-8, MARS), or to assess the likelihood of being or becoming non-adherent (e.g. AE, BMQ). Within some of these instruments, there are questions that can help to identify specific adherence barriers. For example, MMAS-8 asks about forgetfulness – a common cause of non-adherence [4] – and the AE probes the risk of intentional non-adherence by assessing levels of concern about medicines [11]. The BMQ explores specific or general beliefs about medicines, which have been found to be associated with non-adherence behaviour [13–15]. However, none of these tools was designed to screen for a broad range of actual and potential modifiable impediments to adherence.

There are at least two currently available self-reporting tools that can screen for adherence barriers in patients with CV disease: one evaluated adherence in patients taking at least three medicines including enalapril or captropril, and the second focused on patients with hypertension [16, 17], but these have important limitations with regard to structure, the setting in which they were used and their length. We are not aware of any tools that screen for modifiable barriers to adherence in a post MI setting.

We therefore designed a self-reporting tool for identifying modifiable barriers to adherence among patients prescribed CV SPM in normal clinical practice. This instrument, known as the My Experience of Taking Medicines (MYMEDS) questionnaire, was derived from a wide-range literature review, expert input, and an extensive analysis of adherence behaviour among over 500 patients with coronary artery disease [18–20]. The tool was used in clinical practice as part of an innovative post MI medicines and risk optimisation programme which was highly praised by our patients [21]. With the help of MYMEDS we were able to address individual patient issues round SPM, reduce patient concerns about their medications, and ultimately lowering rates of non-adherence by up to 70% at 3–6 months post-clinic [21].

Here, we describe the process of developing MYMEDS, the structure of the finalised tool, and the adherence data and patient feedback from 204 consecutive post-MI subjects who completed an evaluation based on MYMEDS.

## Methods

### Development of the MYMEDS questionnaire

In designing MYMEDS, key principles for the development of adherence scales were followed, including consideration of the administration length of the tool, reliability and validity, ability to detect adherence barriers, the type of non-adherence detected (intentional vs unintentional), transferability, generalisability, ability to assess beliefs about medicines, and the usefulness of the information it provides to support patients and optimise their medicines in the context in which it is validated (coronary artery disease) [22, 23].

Potentially modifiable factors often associated with non-adherence to SPM were initially identified based on a literature review. This was followed by an analysis of our own patients, and these data have been published elsewhere [18–20]. Among many barriers to adherence with SPM identified, the most common were forgetfulness, worry that medicines could do more harm than good, feeling inconvenienced about medicines taking, and not being convinced of the benefit of medicines. Practical barriers were also significantly associated with non-adherence.

Following on from this work, representatives of key stakeholder groups were consulted – including two cardiologists, three cardiac pharmacists, and two cardiac nurses – and a draft adherence assessment questionnaire was then developed. The key focus was to make the tool as comprehensive as possible while also keeping it concise and practical. The draft questionnaire included domains on the way patients took their medicines and the regimens adopted, knowledge about individual SPMs, overall satisfaction and understanding of SPMs, concerns about SPMs, practicalities when taking SPMs, and self-reported adherence to individual SPMs. This draft version was user tested with a pilot group of 10 patients from our *Cardiology Patient and Public Involvement Group*. After completing the tool, we held a pilot group discussion focusing on the overall structure of the questionnaire, any missing dimensions, if any, and the tool’s usability – in particular, aspects of consistency of understanding of questions, readability, length, and repetitiveness. Based on their feedback, the questionnaire was adjusted and then given back to the pilot group for any final comments before it was finalised for use.

The tool was named ‘My Experience of Taking Medicines’ (MYMEDS) to reassure patients that they could volunteer information about their medicines-taking behaviour without being judged.

### Structure and use of the MYMEDS questionnaire

As recommended by the UK National Institute for Health and Care Excellence [22], the MYMEDS questionnaire opens with a brief statement designed to make the patient feel comfortable sharing their experiences: “Taking medicines is an experience that differs from one patient to another. In order for us to support you and provide the help you need, we would like to know how you are managing with your medicines, what questions you have, and any concerns that you need addressing.”

The questionnaire itself is composed of six simple-to-complete sections (Table 1). In section 1, patients provide essential contextual information: the medicines that they are taking, when they are administered each day, and their understanding of why they are taking them. This would enable clinicians to understand what and how a patient takes their medicines compared to what is documented in clinical records, and uncover any medicines changes since discharge. This supports better optimisation of medicines as discussed in the Post MI Medicines Optimisation Clinic model [21].

**Table 1:** Summary of the MYMEDS questionnaire

Section	Area of focus	Content	Completion method
1	Current medicines	Medicines being taken, administration times, why the patient takes them	List of medicines; tick when they take them; state why they take them
2	Understanding and satisfaction with medicines	Understanding of why medicines were prescribed, whether the patient is convinced of the importance of these medicines, whether they feel that their medicines are working	Four-point Likert scale (strongly agree, agree, disagree, strongly disagree); free text for comments
3	Concerns about medicines	Level of worry that medicines will cause more harm than good, concern about being on too many medicines, and whether medicines need to be altered because of actual or perceived harm	Four-point Likert scale (strongly agree, agree, disagree, strongly disagree); free text for comments
4	Practical barriers to adherence	Problems with opening medicine packaging or reading labels, swallowing problems, and issues with obtaining repeat prescriptions	Four-point Likert scale (strongly agree, agree, disagree, strongly disagree); free text for comments
5	Fitting medicines into daily routine	Issues with forgetfulness and inconvenience due to taking medicines	Four-point Likert scale (strongly agree, agree, disagree, strongly disagree); free text for comments
6	Adherence to individual SPMs	Adherence levels with each separate SPM being taken	Five-point Likert scale for how often each medicine was taken as prescribed in the past month (all of the time; nearly all of the time; most of the time; about half the time; less than half the time)
Final	Other	Any other issues that the patient wishes to raise	Free text

*MYMEDS* My Experience of Taking Medicines, *SPM* Secondary prevention medicine

Subsequent sections were largely based on Likert scales. The use of such scales rather than binary yes/no responses is generally recommended because it improves the quality of information obtained [22, 24]. For example, the frequency of non-adherence behaviours can be assessed rather than just the presence of non-adherence [24].

Section 2 explores overall understanding and satisfaction with their medicines. Patients are provided with four statements about understanding the reasons why their medicines were prescribed and how well they feel they are working, and are asked to assess their agreement on a four-point Likert scale (strongly agree, agree, disagree, strongly disagree).

Sections 3–5 assess specific modifiable barriers to adherence, using the same four-point Likert scale as in section 2. A free-text box allows patients to specify which medicines their concerns involve.

Section 3 explores three areas of anxiety about medicines (e.g. worry that they will cause more harm than good, or that there are too many of them), section 4 examines four separate practical concerns associated with medicines taking (e.g. swallowing problems and problems getting repeat prescriptions), and section 5 assesses three issues in fitting medicines into patients’ daily routine (e.g. relating to forgetfulness or inconvenience). These issues have been associated with non-adherence in our research and that of others [4, 20, 22, 25], and can potentially be addressed in clinical practice.

Section 6 asks about adherence to each individual SPM over the past month. It is based on a modified version of the SQ tool [9]. In our experience, this tool alone is not sufficiently sensitive to distinguish all non-adherence behaviour but, within the context of the whole MYMEDS questionnaire, it can be helpful in identifying specific problematic medicines [20]. A five-point Likert scale is used in this section of MYMEDS, and patients are considered to be non-adherent if they select any answer other than ‘all of the time’ for adherence to any SPM.

At the end of the questionnaire, patients can write down in free text any other concerns or issues that they wish to raise. We have found that when patients are given an opportunity to be heard, they are often keen to share more information about their medicines-taking experience [26].

The study was conducted as part of a new development project to improve post MI medicines and risk optimisation at our teaching hospital [21]. The present study includes consecutive patients who completed the final version of the MYMEDS questionnaire. All had been identified on the cardiology wards after being admitted to hospital with MI, and were then scheduled to attend an outpatient Post MI medicines optimisation clinic designed to optimise secondary prevention medicines, risk factors and address adherence barriers [21]. The questionnaire was mailed out around 4–6 weeks before the clinic appointment and patients were asked to bring it with them for discussion during the consultation. Demographic data were collected as part of that consultation.

Although the primary purpose of MYMEDS is to identify barriers to adherence, regardless of the patient’s actual adherence status, some elements of the questionnaire can be used to identify non-adherence behaviour: section 2d, ‘at least occasionally, I need to alter my medicines on my own to make them work or meet my expectations’; section 3c, ‘I sometimes alter my medicines by cutting back or stopping taking them’; section 4c, ‘I have difficulties or problems getting my repeat prescriptions and would like help ordering them from my GP or pharmacy’; section 5a, ‘I sometimes forget to take my heart medicines’; section 5b, ‘I am finding it difficult to fit one or more of my heart medicines into my daily routine’; and section 5c, ‘I feel inconvenienced/bothered about sticking to all my heart medicines’. Acknowledgement of non-adherence behaviour according to at least one of these six questions was used to generate an estimate of overall rates of non-adherence. This was not validated at this stage as it is not the main objective of MYMEDS.

As the creation of MYMEDS was part of a service development programme in clinical practice, ethics approval was not required as advised by our Research and Innovation department and in line with Health Research Authority guidance and its decision tools. Data collection was conducted in accordance with the Declaration of Helsinki.

### Patient feedback on the MYMEDS questionnaire

After attending the clinic, patients were asked to provide anonymous quantitative feedback about the MYMEDS questionnaire, based on five questions assessed using a four-point scale (strongly agree, agree, disagree, strongly disagree). Qualitative feedback could also be provided using free text, based on the question ‘What was best about the service and what can we improve?’. Only answers related to MYMEDS were included in this analysis.

## Results

### Effectiveness of the MYMEDS questionnaire in revealing adherence barriers

The MYMEDS questionnaire was sent to 270 patients before they attended the medicines optimisation clinic. In total, 66 individuals did not complete the questionnaire, for example because they said they had not received it in the post or because they preferred to discuss relevant issues in person.

The performance of the MYMEDS questionnaire was therefore examined by collating the responses of 204 patients who completed it. The mean age of these individuals was 70.5 years and most were male (*n* = 138; 67.6%) (Table 2). Based on MYMEDS reports, the majority of patients were found to be taking several different classes of SPM, and most had at least two regular SPM administration times per day. There were no significant differences in baseline characteristics between those who completed the questionnaire (*n* = 204) and those who did not (*n* = 66), other than mean age (70.5 ± 10.9 vs 66.2 ± 14.5 years, respectively; *p* = 0.022).

**Table 2:** Patient characteristics

Variable	Baseline value (*N* = 204)
Age, years, mean (SD)	70.5 (10.9)
Sex, n (%)	
Male	138 (67.6)
Female	66 (32.4)
Type of MI, n (%)	
NSTEMI	127 (62.3)
STEMI	77 (37.7)
SPM, n (%)	
Aspirin	190 (93.1)
Other antiplatelet agent	192 (94.1)
Ticagrelor (twice daily)	159 (77.9)
ACE inhibitor / ARB	188 (92.2)
Twice daily dosing	44 (21.6)
Beta-blocker	180 (88.2)
Twice daily dosing	23 (11.3)
Statin	192 (94.1)
SPM administration times,* n (%)	
One regular administration time per day	5 (2.5)
Two regular administration times per day	147 (72.1)
Three regular administration times per day	40 (19.6)
Not completed	12 (5.9)

*N* = 204. *The total number of times the patient had to take a medicine, regardless of the number of medicines taken at the point of administration (i.e. more than one medicine may have been taken at each administration time). *ACE* Angiotensin converting enzyme, *ARB* Angiotensin II receptor blocker, *NSTEMI* non-ST-elevation myocardial infarction, *MI* Myocardial infarction, *SD* Standard deviation, *SPM* Secondary prevention medicine, *STEMI* ST-elevation myocardial infarction

Among those who completed the questionnaire, knowledge about individual SPMs was poor: fewer than half of respondents knew the indications for each of their SPM medicines.

The tool was effective in revealing many important modifiable barriers to SPM adherence (Table 3), which could then be addressed in the medicines optimisation clinic. In particular, 33.2% of patients (*n* = 62/187) were concerned that their SPM would cause them more harm than good, and 43.2% (*n* = 82/190) were concerned about being prescribed too many medicines; 8.2% (*n* = 16/194) reported problems with swallowing medicines, and 7.3% (*n* = 14/191) said that they had problems with opening medicine bottles or packs; 19.7% of patients (*n* = 38/193) agreed that they sometimes forget to take one or more of their medicines, and 13.5% (*n* = 26/193) felt inconvenienced or bothered about sticking to their SPMs.

**Table 3:** Collated results from sections 2–5 of the MYMEDS questionnaire

Statement	Response, n (%)			
	Strongly agree	Agree	Disagree	Strongly disagree
Section 2: Understanding and satisfaction with medicines				
a. I fully understand my heart medicines and why they were prescribed (*N* = 196)	47 (24.0)	95 (48.5)	44 (22.4)	10 (5.1)
b. My heart medicines seem to be working for me (*N* = 183)	49 (26.8)	116 (63.4)	15 (8.2)	3 (1.6)
c. I feel convinced of the importance of all my heart medicines (*N* = 194)	79 (40.7)	102 (52.6)	12 (6.2)	1 (0.5)
d. At least occasionally, I need to alter my medicines on my own to make them work or meet my expectations (*N* = 180)	9 (5.0)	17 (9.4)	64 (35.6)	90 (50.0)
Section 3: Concerns about medicines				
a. I worry that one or more of my medicines will do me more harm than good (*N* = 187)	13 (7.0)	49 (26.2)	87 (46.5)	38 (20.3)
b. I feel concerned about being prescribed too many medicines (*N* = 190)	15 (7.9)	67 (35.3)	74 (38.9)	34 (17.9)
c. I sometimes alter my medicines by cutting back or stopping taking them (*N* = 186)	6 (3.2)	9 (4.8)	89 (47.8)	82 (44.1)
Section 4: Practical issues that may be a barrier to adherence				
a. I have difficulties or problems opening the medicine bottles or blister packs and would like a solution or an alternative (*N* = 191)	2 (1.0)	12 (6.3)	87 (45.5)	90 (47.1)
b. I have difficulties or problems swallowing my medicine(s) and would like a solution or an alternative (*N* = 194)	4 (2.1)	12 (6.2)	88 (45.4)	90 (46.4)
c. I have difficulties or problems getting my repeat prescriptions and would like help ordering them from my GP or pharmacy (*N* = 192)	3 (1.6)	7 (3.6)	96 (50.0)	86 (44.8)
d. I have difficulties or problems reading the label on the medicines bottle or box and would like a solution or alternative (*N* = 191)	2 (1.0)	5 (2.6)	100 (52.4)	84 (44.0)
Section 5: Fitting medicines into daily routine				
a. I sometimes forget to take my heart medicines (*N* = 193)	4 (2.1)	34 (17.6)	78 (40.4)	77 (39.9)
b. I am finding it difficult to fit one or more of my heart medicines into my daily routine (N = 194)	5 (2.6)	17 (8.8)	89 (45.9)	83 (42.8)
c. I feel inconvenienced/bothered about sticking to all my heart medicines (N = 193)	5 (2.6)	21 (10.9)	90 (46.6)	77 (39.9)

*MYMEDS* My Experience of Taking Medicines

Overall, 193 patients (94.6%) completed at least one question in all four domains of MYMEDS relating to adherence barriers (sections 2–5). Of these, 33.2% of respondents (*n* = 64) had adherence barriers relating to only one domain, 25.4% (*n* = 49) had at least one barrier in two domains, 16.1% (*n* = 31) in three domains, and 3.6% (*n* = 7) in four domains. Across all domains, 29.0% of respondents (*n* = 56) had one adherence barrier, 22.3% (*n* = 43) had two, 13.5% (*n* = 26) had three, 7.8% (*n* = 15) had four, and 5.7% (*n* = 11) had five or more barriers. The mean number of barriers per patient was 1.8 ± 1.5.

**Fig. 1:**
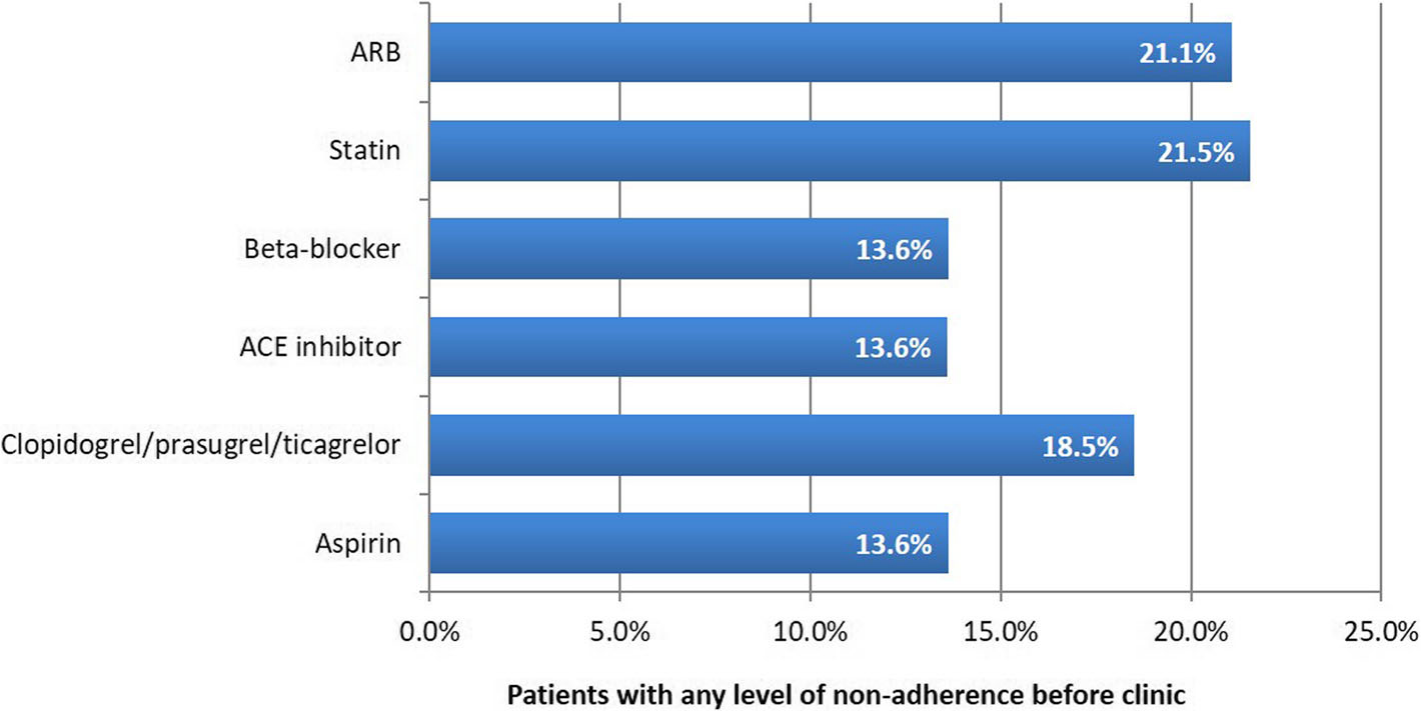
Non-adherence to individual SPMs (MYMEDS section 6). Percentages were calculated using the total number of patients who took each medicine as the denominator (ARB, *N* = 38; statins, *N* = 181; beta-blocker, *N* = 169; ACE inhibitor, *N* = 140; clopidogrel / prasugrel / ticagrelor, *N* = 173; aspirin, *N* = 176). ACE, angiotensin converting enzyme; ARB, angiotensin II receptor blocker; MYMEDS, My Experience of Taking Medicines; SPM, secondary prevention medicine

The classes of medication most commonly associated with non-adherence were statins (*n* = 39/181; 21.5% of patients), angiotensin II receptor blockers (ARBs; *n* = 8/38; 21.1%), and antiplatelet agents (clopidogrel / prasugrel / ticagrelor; *n* = 32/173; 18.5%) (Fig. 1). Non-adherence was also described with beta-blockers (*n* = 23/169; 13.6%), angiotensin converting enzyme inhibitors (*n* = 19/140; 13.6%), and aspirin (*n* = 24/176; 13.6%). Among those who reported non-adherence to individual SPM, 51.3% (n = 23/44) said that they were non-adherent to only one drug.

Overall, 86 patients (42.2%) used the free text box at end of the questionnaire to provide a total of 154 additional comments. Key themes are summarised in Table 4. Almost a third of comments were related to side effects (e.g. experiencing specific side effects, suspecting particular symptoms were caused by SPMs, or concern about possible future side effects).

**Table 4:** Key themes raised in the free-text section of the MYMEDS questionnaire

Theme	Details
Side effects	Mostly relating to experiencing specific side effects, suspecting that they were caused by one or more medicines, or concern about possible side effects (e.g. headaches, anxiety, cold hands, panic attacks, dizziness, cramps, bruising, feeling tired, breathlessness)
Pharmaceutical form	Issues around different formulations, generics, size of tablets, dissolving tablets, and desire for easier-to-swallow formulations
Administration	Issues around the time of the day to take medicines, taking medicines together, whether ‘twice a day’ mean 12 h apart
Medicines interactions	Queries about drug–drug and drug–food/drink interactions, and concerns about taking too many medicines for the same thing (e.g. too many blood pressure-lowering medicines)
Rationale for medicines	Questions on why to take certain medicines when key parameters were within the target range (e.g. why take hypertension medication when blood pressure is well controlled, why take a statin when blood cholesterol is low)
Service issues	In particular, reporting service problems (e.g. with obtaining repeat prescriptions)
Requests for further information	In particular, more information about side effects, or about the length of time on medicines (e.g. how long time to take a high-dose statin, when to stop a second antiplatelet, extending dual antiplatelet therapy)
Role of carer	Notes on the roles of their carers in taking medicines (e.g. my family support me, my daughter sorts out my medicines)

*MYMEDS* My Experience of Taking Medicines

### Overall adherence

Acknowledgement of non-adherence behaviour according to at least one of six indicator questions in MYMEDS (sections 2d, 3c, 4c, and 5a–c; Table 3) was used to generate an estimate of overall rates of non-adherence. In total, 42.5% of patients (*n* = 82/193) who responded fitted these non-adherence criteria.

### Patient feedback on MYMEDS

A total of 131 patients provided feedback on MYMEDS, and this showed that the questionnaire was well received (Table 5). There was near unanimous accord that it helped them to think about their medicines (*n* = 127; 96.9% agreed), and that it enabled them to raise any questions or concerns they had about their medicines during the subsequent consultation (*n* = 128; 97.7%). In addition, almost all of the patients who provided feedback agreed that the questionnaire was simple and clear (n = 128; 97.7%), and that the length was acceptable (*n* = 130; 99.2%).

**Table 5:** Patient feedback on the MYMEDS questionnaire

Statement	Agree, n (%)	Disagree, n (%)
The questionnaire helped me think about my medicines	127 (96.9)	4 (3.1)
The questionnaire was simple and clear	128 (97.7)	3 (2.3)
The length of the questionnaire was acceptable	130 (99.2)	1 (0.8)
The questionnaire was helpful in making me think about issues related to my medicines before visiting the clinic	130 (99.2)	1 (0.8)
The questionnaire helped me to raise any concerns I had about my medicines during the consultation	128 (97.7)	3 (2.3)

*MYMEDS* My Experience of Taking Medicines. *N* = 131

Furthermore, feedback from individual patients suggested that the MYMEDS questionnaire was straightforward to complete (“It was easy to understand, and easy to answer”), facilitated improved understanding of individual therapies (“It made me think what I was taking rather than just accepting the meds given”; “I found the questionnaire helpful – in that it encouraged me to find out and understand more about my medications”), and aided medicines optimisation (“The questionnaire helped the consultant easily find some discrepancies about my medicines and altered them accordingly”). Some suggestions were also made for improving the overall service, particularly relating to transactional aspects such as waiting time and environment; this feedback has fed into the ongoing optimisation of the clinic. There were very few negative or improvement comments about MYMEDS. For example: one patient commented “.. expect some elderly patients to require some assistance to complete”. Indeed, as with other questionnaires, some elderly patients may need help to complete MYMEDS. The clinic invitation letter acknowledges that some patients may need the help of their carers to complete the questionnaire.

## Discussion

This study demonstrated the capacity of the MYMEDS self-reporting questionnaire to identify actual and potential barriers to adherence to SPM – that could be addressed in clinical practice – in 204 consecutive post-MI patients. Moreover, user feedback was highly positive, with nearly all patient agreeing that the tool was simple to use and facilitated discussion of issues and concerns around medicines taking. Levels of patient engagement were high, with almost half of respondents using the free-text section at the end of the questionnaire to share additional issues and concerns relating to their SPM.

Importantly, the tool was not used in isolation. Instead, completed questionnaires were carefully reviewed during a subsequent outpatient medicines optimisation clinic designed to address the barriers identified [21]. At our centre, this clinic follows a multidisciplinary format: patients who need no further non-pharmacological interventions see the consultant cardiology pharmacist (with the option of a review by a cardiologist if needed), while patients requiring further interventions such as staged percutaneous coronary intervention see a cardiologist (with the option of referral to the consultant pharmacist if needed). Consultations typically last around 20 min, but individuals with more complex issues – such as known or elevated risk for poor adherence – can attend an enhanced session that lasts around 45 min. All consultations take a patient-centred approach, using the completed MYMEDS questionnaire as a basis for optimisation of SPM regimens and discussion of adherence issues. A full management plan is then agreed, including key action points for the patient (or carer) and their healthcare team. This model has been successful in optimising SPM selection and dosing, decreasing patient concerns about their medications, and ultimately lowering rates of non-adherence by up to 70% at 3–6 months post-clinic [21]. Readmission rates have also fallen [21].

In this model, patients have access to both a consultant cardiology pharmacist and a cardiologist, but most are seen only by the pharmacist. This opens up places at the cardiology outpatient clinic and improves overall capacity. Moreover, a recent analysis of data from eight systematic reviews of adherence solutions found that interventions provided by a multidisciplinary team may be more successful in improving adherence compared to those delivered by physicians alone [27].

Use of the MYMEDS questionnaire has played a key role in making the clinic more patient-centred by highlighting individual concerns and building confidence that the consultation will focus on addressing these issues. The overall clinic model has helped to facilitate shared decision making [21], in alignment with current guidelines for improving adherence and optimising medicines taking [22, 28].

Importantly, the MYMEDS questionnaire focuses on actual and potential adherence issues that can be *modified* in clinical practice. Much of the research into the causes of non-adherence has assessed fixed factors or those that cannot be modified in clinical practice, such as sociodemographic and financial status, gender, age, and disease type and severity [4, 29]. The impact of these factors has proved to be inconsistent. By focusing on a wide range of modifiable factors, with a known impact on adherence [4, 20, 22, 25], the MYMEDS questionnaire can be used to start a personalised conversation that puts the patient in control and provides an adaptable action plan for overcoming specific barriers.

The issues identified in the current cohort of patients included high rates of concern that SPMs could be harmful (33.2%) or overprescribed (43.2%), practical issues with swallowing medicines (8.2%), opening packaging (7.3%) or accessing repeat prescriptions (5.2%), forgetfulness (19.7%), and concerns about inconvenience (13.5%). These data are broadly in line with previous work on the underlying causes of non-adherence to CV medicines [20, 30, 31]. Furthermore, all of these issues can potentially be mitigated through straightforward, targeted interventions, such as patient education, adjustments to the treatment plan, and/or enhanced support from friends and family. For example, patients with problems swallowing medicines can be switched to a different formulation or doses can be split, and patients with concerns about harmful effects can be listened to, educated and reassured about specific medicines.

The single question approach to assess adherence in section 6 of the tool was previously validated in various studies [9, 32]. Our work shows that this form of adherence assessment tends to have low sensitivity and high specificity compared to MMAS-8 validated tool [20, 21, 32]. However, the modification of the tool was useful in identifying individual SPM that patients had issues with.

The intention in developing the MYMEDS tool was to identify barriers to adherence rather than to assess specifically whether the patient was adherent or not. However, apart from section 6, some other elements of the questionnaire do identify non-adherence behaviour. Based on these, we estimated that around 42.5% of patients appeared to be non-adherent. This aligns with previous data on adherence with SPM in this setting [1]. This aspect of the tool needs to be validated in the future.

Regarding specific SPMs, adherence problems were most common with statins, ARBs and antiplatelet agents. Non-adherence was usually selective: many patients were non-adherent to one medicine while adhering to all of the others. This aligns with our previously published experience [20]. Once issues were identified at an individual level, they were often easily resolved, and we have discussed some of the relevant strategies in previous publications [20, 21]. For example, some patients had safety concerns about statins, which could typically be alleviated through discussion and education and/or by switching to another statin. Others were non-adherent to a statin because it was the only medicine they took at night, and adherence improved once it was moved to morning administration. Indeed, an important revelation from MYMEDS was how many patients had two or even three different medicine administration times each day, and how often this was associated with easily solvable adherence issues. For example, some patients took an evening dose of ticagrelor with dinner and took a statin separately before going to bed (due to an instruction to ‘take at night’). Adherence could often be improved by advising them to take both at the same time or by offering a once-daily alternative to ticagrelor, if appropriate.

There are other tools that have been developed to assess the extent of and underlying reasons for non-adherence [16, 17]. For example, Svarstad and colleagues developed the Brief Medication Questionnaire (BrMQ) based on the Health Collaboration Model [16, 33]. The BrMQ explores the patient’s medicines regimen, asking them to list each one and answer specific questions covering several domains which are similar to MYMEDS. While this approach may provide comprehensive information, it can be time consuming if the patient is taking many medicines. In the first iteration of MYMEDS, SPMs were listed separately and respondents were questioned on each. However, the pilot group of patients disliked this format and considered it too lengthy. Thus, in the final version, patients were given general questions and then identified which medicines were problematic. For example, they were asked about forgetting to take medicines, and could specify which ones in a free-text box. MYMEDS also covers some issues that the BrMQ does not. For example, it actually enables identification of actual administration times (rather just how many times a day) and asks about difficulties with swallowing medicines. In addition, MYMEDS ends with an open question that allows patients to share their thoughts and medicines-taking experiences. This was often a particularly useful instrument for discussion in the subsequent clinic.

More recently, Voils and co-workers developed a self-report measure that assesses both the extent of and reasons for medication non-adherence among patients with hypertension [17]. This tool is another excellent attempt to understand self-reported reasons behind non-adherence. The instrument first asks the patient to report the extent of non-adherence and then focuses on rating potential underlying reasons for non-adherence. By contrast, MYMEDS is deployed to identify actual or potential barriers irrespective of current adherence levels, and indeed the measuring of adherence itself is a secondary goal. This is a crucial difference because an adherent patient should also be supported if they are faced with a barrier which is making their medicines-taking experience more challenging. In addition, an adherent patient may become non-adherent in the future if the barrier to adherence is not resolved. MYMEDS is also less lengthy to complete, and includes a number of potential barriers not listed in the Voils tool (e.g. difficulties accessing repeat prescriptions or opening packaging).

Although the MYMEDS questionnaire was specifically used in post-MI patients in the current study, there is no theoretical reason why it could not be used with any other individual taking preventive CV medicines. Indeed, the near unanimous agreement that MYMEDS was both valuable and easy to use suggests that it could be usefully deployed in many other patients with CV disease – albeit that section 6 might need to be modified to reflect appropriate classes of medicines. In addition, while a paper version was used during the development and testing of the tool, in the future we will be exploring electronic versions on various platforms.

We should acknowledge some limitations of the present study. First, MYMEDS is based on patient self-reporting, and therefore has potential memory and social desirability biases that may lead to over-estimation of adherence. However, there is no reason to believe that *non-adherence* behaviours identified via MYMEDS are inaccurate. Second, MYMEDS was not validated against another adherence tool or prescription refill data. However, estimation of overall adherence rate was not the main purpose of developing the questionnaire. Furthermore, when validating a self-reported tool, the usual concerns are around lack of sensitivity and under-estimation of non-adherence, which does not seem the case with MYMEDS. Nonetheless, formal validation might be an important future undertaking. Third, the present assessment of the questionnaire was based on a single-centre, non-comparative study design. Undoubtedly, a prospective, multicentre, randomised controlled trial of the use of MYMEDS to improve adherence (and patient outcomes) would be valuable. Finally, there is a possibility of reporting bias in the patient feedback on MYMEDS. Because this was completely anonymised, the characteristics of those who did and did not provide feedback cannot be compared. However, the response rate was high, which suggests that a reasonable cross-section of patients may have responded.

## Conclusions

Overall, this study shows that MYMEDS is a simple-to-use, practical tool that can successfully identify many actual and potential modifiable barriers to SPM adherence. It can easily be rolled out in clinical practice to direct consultations and improve their patient focus.

## Data Availability

The original datasets used and/or analysed during the current study are available from the corresponding author on reasonable request.

## Abbreviations

ACE: Angiotensin converting enzyme
AE: Adherence estimator
ARB: Angiotensin II receptor blocker
BMQ: Beliefs about medicines questionnaire
BrMQ: Brief medication questionnaire
CV: Cardiovascular
MARS: Medication adherence report scale
MI: Myocardial infarction
MMAS-8: Eight-item Morisky medication adherence scale
MYMEDS: My experience of taking medicines
NSTEMI: Non-ST-elevation myocardial infarction
SD: Standard deviation
SPM: Secondary prevention medicine
SQ: Single question tool
STEMI: ST-elevation myocardial infarction
